# Fabrication of Glutaraldehyde Vapor Treated PVA/SA/GO/ZnO Electrospun Nanofibers with High Liquid Absorbability for Antimicrobial of *Staphylococcus aureus*

**DOI:** 10.3390/nano13050932

**Published:** 2023-03-03

**Authors:** Yi-Hsin Chien, Meng-Tzu Ho, Chin-Hsign Feng, Jung-Hsign Yen, Yi-Chan Chang, Chih-Sheng Lai, Rong-Fuh Louh

**Affiliations:** 1 Department of Materials Science, Feng Chia University, Taichung 40724, Taiwan; 2 Division of Plastic Surgery, Taichung Veterans General Hospital, Taichung 40705, Taiwan

**Keywords:** electrospinning, polyvinyl alcohol, sodium alginate, graphene oxide, zinc oxide, glutaraldehyde vapor, nanohybrid membranes, antibacterial properties, water retention ratio, surgical dressings

## Abstract

In this study, we aim to develop organic–inorganic hybrid nanofibers containing high moisture retention and good mechanical performance as an antimicrobial dressing platform. The main theme of this work focuses on several technical tasks including (a) the electrospinning process (ESP) to produce organic polyvinyl alcohol/sodium alginate (PVA/SA) nanofibers with an excellent diameter uniformity and fibrous orientation, (b) the fabrication of inorganic nanoparticles (NPs) as graphene oxide (GO) and ZnO NPs to be added to PVA/SA nanofibers for enhancement of the mechanical properties and an antibacterial function to *Staphylococcus aureus (S. aureus)*, and then (c) the crosslinking process for PVA/SA/GO/ZnO hybrid nanofibers in glutaraldehyde (GA) vapor atmosphere to improve the hydrophilicity and moisture absorption of specimens. Our results clearly indicate that the uniformity nanofiber with 7 wt% PVA and 2 wt% SA condition demonstrates 199 ± 22 nm in diameter using an electrospinning precursor solution of 355 cP in viscosity by the ESP process. Moreover, the mechanical strength of nanofibers was enhanced by 17% after the handling of a 0.5 wt% GO nanoparticles addition. Significantly, the morphology and size of ZnO NPs can be affected by NaOH concentration, where 1 M NaOH was used in the synthesis of 23 nm ZnO NPs corresponding to effective inhibition of *S. aureus* strains. The PVA/SA/GO/ZnO mixture successfully performed an antibacterial ability with an 8 mm inhibition zone in *S. aureus* strains. Furthermore, the GA vapor as a crosslinking agent acting on PVA/SA/GO/ZnO nanofiber provided both swelling behavior and structural stability performance. The swelling ratio increased up to 1.406%, and the mechanical strength was 1.87 MPa after 48 h of GA vapor treatment. Finally, we successfully synthesized the hybrid nanofibers of GA-treated PVA/SA/GO/ZnO accompanied with high moisturizing, biocompatibility, and great mechanical properties, which will be a novel multi-functional candidate for wound dressing composites for patients receiving surgical operations and first aid treatments.

## 1. Introduction

In recent years, the versatility and applicability of nanofiber-based products have been realized in a wide range of regions such as (a) biomedical for tissue engineering of bones or scaffolds in the orthopedics-related study, wound composite therapy, and media for drug delivery applications [1,2,3]; (b) electronic materials for electronic packaging, sensors, and fuel cells [4,5,6], and (c) industrial for filter materials, thermal insulation materials, high-performance cleaning cloths, reinforced composites, and functionally protective materials [7,8]. Generally, there are a number of common methods of nanofiber preparation, including drawing, template synthesis, phase separation, self-assembly, and electrospinning. The merits of the electrospinning process consist of simple equipment and process, high yield, cost-effectiveness, being applicable for the wide choice of polymer types, controllability of fiber size uniformity and nanofiber orientation, as well as the porosity control for the texture of membrane via experimental parameters [9,10].

Generally, the involvement of unstable jet morphologies associated with the electrospinning process depends on several operating parameters such as pump flow rate, electric field strength, needle aperture size, the surface charge density of the jet, collection distance combined with environmental conditions such as operating temperature, humidity, airflow, etc. [11]. Moreover, the size of electrospun fibers increases by raising the electrospinning solution’s viscosity or pump speed, the polymer agent’s molecular weight, the applied electrical field, and the humidity of the operating environment. An increase in collection distance and surface charge density or electrical conductivity of the electrospinning solution can be effectively tuned to reduce the size of electrospun fibers. Interestingly the surface of electrospun fibers is found to be associated with a great number of pores with bigger pore size under high humidity of the operating environment due to water condensation [12].

The qualified wound dressing needs to require features such as air permeability, moisture absorption, water retention, non-stickiness, acceptable mechanical strength, antibacterial ability, and biocompatibility [3]. Therefore, new types of hybrid nanofibers recently have attracted considerable attention in the area of wound dressings production for medical treatment purposes [13,14,15]. Accordingly, polyvinyl alcohol (PVA), sodium alginate (SA), poly(ε-caprolactone) (PCL), poly(glycolic acid) (PGA), and cellulose are the widely accepted polymers to fabricate electrospun nanofibers as medical surgical dressing substrates due to their excellent hygroscopicity, nontoxicity, and degradation property [16,17]. Thus, the blend and co-polymer fabrication become attractive by aptly adjusting materials properties such as hygroscopicity, toughness, mechanical strength, cellular affinity, and biodegradability. Sardou et al. reported that skin tissue regeneration shows high cell attachment and proliferation affinity by polymer blending of PCL and gelatin electrospun nanofibers [18]. Ebrahimi et al. synthesized electrospun PCL homopolymer and PCL-PEG-PCL triblock copolymer-based nanofibers for tissue engineering applications accompanied by hydrophilicity, biocompatibility, non-toxicity, non-antigenic, and non-immunogenic characteristics [19]. In this context, a polymer combined with an inorganic nanoparticle such as hydroxyapatite (HA), magnesium oxide (MgO), zinc oxide (ZnO), graphene, and silver nanoparticles (Ag NPs) to form the hybrid nanofibers corresponds to a diverse range of scaled scaffolds, mechanical, and structural integrity [20]. Rijal et al. [21] prepared PCL/MgO and PCL/chitosan (CS)/MgO-based composite nanofibrous membranes through an electrospinning process. The tensile strength and Young’s modulus were enhanced during the increase in MgO NPs concentration. Moreover, the presence of salt ions in the electrospinning solution results in a reduction in the fiber diameter due to raising the surface charge density of the polymer jet leading to the stretching of fibers. In addition, the Ag and ZnO NPs are commonly employed as antimicrobial agents, and the *S. aureus* study of PCL-Ag composite nanofibers showed an increase in the inhibition zone with higher Ag NPs concentration [22].

**Scheme 1:**
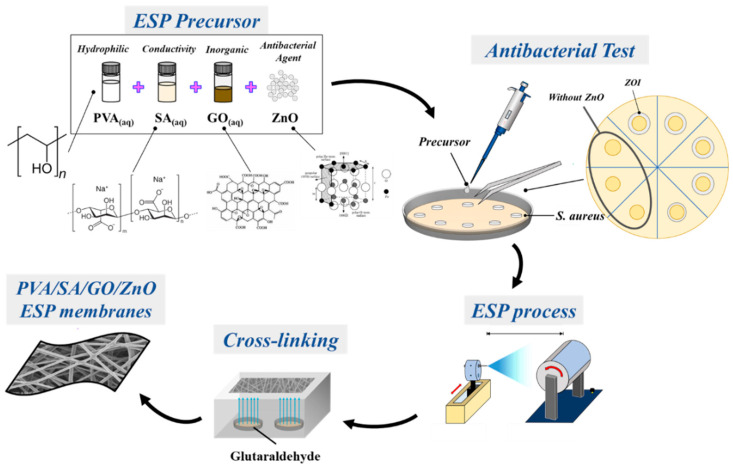
The synthesis flowchart of GA-treated PVA/SA/GO/ZnO electrospun membranes against *Staphylococcus aureus* (*S. aureus*).

This study mainly aimed to investigate the binary composite fibers containing organic portions of PVA and SA and inorganic portions of GO and ZnO NPs by using the electrospinning process. Due to the overall polymerization degree of PVA reflecting high viscosity range, it is facile and easy to dissolve quickly when encountering wet conditions. Thus, a natural biopolymer SA extracted from brown algae was selected as a constituent for our dressing design in order to maintain the function of water retention and dressing structure [23]. Interestingly, glutaraldehyde (GA) was frequently utilized as a crosslinking agent in PVA-related processing since the in situ crosslinking step generates chemical bonds between different molecular chains, resulting in a stable and insoluble three-dimensional network structure with improved mechanical strength in aqueous conditions [24,25]. Therefore, the use of GA-treated PVA/SA/GO/ZnO electrospun membranes of nanofibers as wound dressings presents promising antimicrobial performance to *S. aureus* (as seen in Scheme 1).

## 2. Materials and Methods

### 2.1. Materials

Polyvinyl alcohol (PVA, 98.0–98.8%) was purchased from Acros Organics (Geel, Belgium). Sodium alginate (SA) and glutaraldehyde were procured from Sigma-Aldrich (St. Louis, MO, USA). Natural graphite powder (>99.7%) was acquired from the Great Carbon Company (Taichung City, Taiwan). Sodium nitrate (NaNO_3_, 99.0%), potassium manganate (KMnO_4_, 99.5%), and hydrogen peroxide (H_2_O_2_, 30%) were acquired from Showa Chemical Company (Tokyo, Japan). Hydrochloric acid (HCl and sulfuric acid (H_2_SO_4_, 98%) were made by Shimakyu Chemical Company (Osaka, Japan). Zinc nitrate, 6-hydrate, and sodium hydroxide (NaOH) were sourced from J.T. Baker (St. Paul, MN, USA). Ethanol was acquired from the Echo Chemical Company (Kaohsiung City, Taiwan). All reagents were used without further purification.

### 2.2. Synthesis of PVA/SA Nanofiber

The synthesis of PVA/SA nanofiber was performed following the previous literature with slight modifications [26]. In preparation for the PVS/SA electrospinning (ESP) precursor agent, the PVA powder was dissolved into DI water (5, 6, 7, 8 wt%) and stirred at 200 rpm for 2 h at 90 °C. The nanofiber precursor could be prepared by mixing the PVA (5, 6, 7, 8 wt%) and the 2 wt% SA solution at room temperature. The precursor was filled in the syringe for ESP equipment and formed the PVA/SA composite nanofibers under settings of 15 kV voltage, 15 cm working distance, and 0.5 mL/h flow rate. That adjustment of processing parameters could be attentively controlled by the viscosity of the PVA/SA precursor to achieve the best conditions for the ESP nanofibers.

### 2.3. Synthesis of Graphene Oxide (GO)

The GO is produced using a slightly modified Hummers’ method [27]. The pure graphite of 1.2 g and 0.38 g sodium nitrate were added to 31.2 mL of sulfuric acid in a serum bottle and stirred in an ice bath (0 °C) for 30 min. KMnO_4_ of 7.2 g was slowly added into the previous solution and kept at 0 °C to prevent an intensive exothermic reaction. While stirring in a 35 °C water bath, the solution color then gradually turned from dark green to dark brown. While the graphite was progressively oxidized, the spacings between the graphite layers subsequently expanded. The previous solution was gently added into 55.2 mL of deionized water and heated from 35 °C to 98 °C under stirring for 8 min and later cooled for another 15 min at room temperature. The above dark brown liquid was added to 120 mL of DI water and 4 mL of hydrogen peroxide (H_2_O_2_, 30%) as a strong oxidizing agent, and the solution finally turned light brown color.

The solution obtained by the Hummers’ method was washed by centrifugation at high speed (10,000 rpm) for 20 min, while a 5% HCl aqueous solution was added in the next step for centrifugation and repeated four times in a row to reduce sulfate sedimentation in the product and by-products. The precipitation was washed later with DI water at high centrifugation speed (10,000 rpm) for 20 min and repeated for four cycles, then it was agitated with an ultrasonic homogenizer for 15 min. After collecting the dark brown solid remains in the lower layer of the centrifuge tube and drying in a vacuum oven (at 60 °C), the GO nanopowders with high oxygen content were obtained.

### 2.4. Synthesis of PVA/SA/GO Nanofiber

The sample of PVA/SA/GO nanofibers was made by the mixture of the precursor of 7 wt% PVA, 2 wt% SA, and diverse addition of aqueous GO solution (0.3, 0.5, 0.7, and 1 wt%) under 15 kV voltage, 15 cm working distance, and 0.5 mL/h flow rate. The conductivity of GO might influence the stability of the spinning jet behavior and cause a non-uniform fiber diameter or formation of a bead-like structure. To discuss the influence of the different concentrations of GO, the ESP, the TEM, SEM, and tensile testing machines were used to analyze the morphology and the mechanical strength of the PVA/SA/GO nanofibers.

### 2.5. Synthesis of ZnO Powder by Precipitation Method

The 0.5 M Zn(NO_3_)_2_ solution (100 mL) was stirred for 1 h at 70 °C until it was completely dissolved. The size of ZnO NPs could be adjusted by different NaOH concentrations. The Zn(NO_3_)_2_ solution was poured into a burette and then dropwise added to the NaOH solution through vigorous stirring [28]. The mixture gradually turned milky white. The mixture was consecutively stirred for 2 h and settled for another 24 h. After that, the mixture was filtered, and the precipitation was washed with DI water and ethanol twice to remove the by-products. Thereafter, the precipitates were dried in an oven at 70 °C and calcined at 400 °C for 3 h for ZnO NPs generation. Correspondingly, the ZnO NPs were used as one gradient of ESP precursor to produce the PVA/SA/GO/ZnO nanofiber. Note that the excessive ZnO NPs addition would destroy the fiber morphology and affect the fiber diameter evident through TEM and SEM analysis.

### 2.6. PVA/SA/GO/ZnO Antibacterial Solution Test

According to the disk diffusion method, the PVA/SA/GO/ZnO solution was dispensed in a paper disk (1 cm dia.) by micropipette to carry out the antibacterial experiment in *S. aureus.* The *S. aureus* strain was sampled by a cotton swab and spread evenly through three different directions on the surface of the medium by a rotating 60° sequence each time. Subsequently, the paper disks containing PVA/SA/GO/ZnO solution were put on the agar medium with *S. aureus* and then incubated for 24 h to observe the growth of the bacterium. All the bacteriostatic experiments of our specimens were collaboratively conducted in the qualified laboratory of the Laboratory Division of Taichung Veterans General Hospital for examining the dimensions of the bacteriostatic rings developed in the petri dish.

### 2.7. PVA/SA/GO/ZnO Vapor Crosslinking with Glutaraldehyde (GA)

These polymers would crosslink by vapor GA at room temperature, preventing them from easily dissolving in water. First, 5 mL of GA and PVA/SA/GO/ZnO nanofiber were taken in a closed container [29]. Normally, the spontaneously generated GA vapor at the ambient condition interactions between the –OH group (from PVA molecule) and the –CHO group (from GA molecule), the crosslinking reaction Equation (1) was shown with graphical expression as follows. The Fourier transform infrared spectroscopy (FTIR), tensile testing, water contact angle testing, swelling testing, and water retention were used for evaluation.



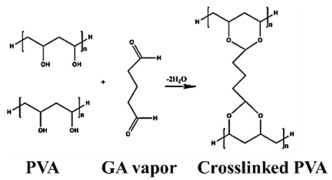

(1)


### 2.8. Swelling Percentage and Water Retention

The crosslinked ESP fiber was cut into 5 cm × 5 cm pieces and its dry weight was measured. The piece was swelled with phosphate-buffered saline (PBS) for 2 h. After swelling, we wiped the surface gently with wipe paper to remove excess water and weighed the sample.

(2) $$\mathrm{Swelling}\;\mathrm{percentage}\;(\%)=\frac{W_{S}-W_{o}}{W_{0}}\times 100$$

where Ws is the weight after swelling and Wo is the weight of the sample before swelling.

The water retention was the ratio between the weight of the sample after swelling in the PBS at 37 °C for 24 h (Ws) and the weight of the sample dried in the oven at 40 °C and detected by time (Wi).

(3) $$\mathrm{Water}\;\mathrm{retention}\;(\%)=\frac{W_{i}}{W_{S}}\times 100$$

where W_s_ is the weight after swelling, and W_i_ is the weight of the sample dried in the oven at different times.

### 2.9. Characterization

The microstructure of the composite nanofibers structure was examined by a field emission scanning electron microscope (FE-SEM, Hitachi, S-4800, Tokyo, Japan). The lattice structure and grain size on the composite fiber and nanopowders were analyzed by multipurpose X-ray thin-film micro-area diffractometer (Bruker, D8 Discover, Billerica, MA, USA). The electrospun fibers containing GO and ZnO NPs were inspected by transmission electron microscopy (JEOL, JEM 1400, Tokyo, Japan). FTIR spectra were detected by Fourier transform infrared spectroscopy (Perkin Elmer, Spectrum One, Waltham, MA, USA). The Raman spectra were examined in the range from 500–2000 cm^−1^ at 300 K with the excitation source at 532 nm wavelength. The viscosity of the PVA solution was recorded by a viscometer (Brookfield, DV-ΙΙ+ Pro, USA). The mechanical strength was measured followed by ASTM D882. The wettability of the sample would present as the contact angle detected by a surface tension measurement apparatus (First Ten Angstroms, Inc., model FTA 125, Portsmouth, VA, USA).

## 3. Results and Discussion

### 3.1. PVA/SA Electrospun Nanofibers

**Figure 1:**
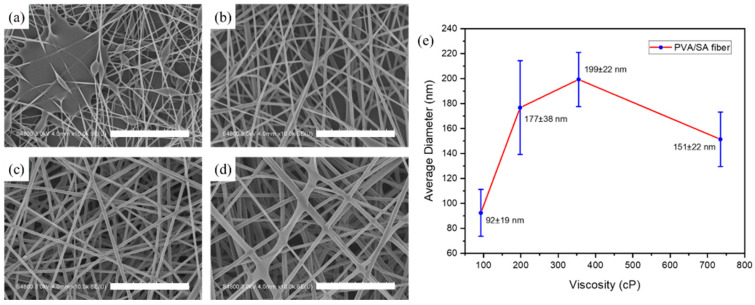
The SEM images of PVA/SA nanofiber were prepared by different precursors containing PVA (5, 6, 7, and 8 wt%) and 2 wt% SA with different viscosities: (**a**) 93 cP; (**b**) 198 cP; (**c**) 355 cP; (**d**) 734 cP. (**e**) the diameter of PVA/SA nanofiber followed by different viscosity. (The scale bar in the SEM image was 5 μm).

The different types of PVA/SA nanofibers were fabricated via ESP technology with mass percentage concentrations (wt%) of PVA/SA precursor, applied voltage, and working distance adjustment. To our observation, the predominant factor in the ESP process was the viscosity of the precursor; for example, the bead structure or ribbon-like structure occurred during the inappropriate viscosity. To optimize the suitable viscosity, the PVA/SA precursor was prepared by different weight percent (wt%) of PVA (5, 6, 7, and 8 wt%), fixed 2 wt% SA, 15 kV voltage, and 15 cm working distance. The viscosity of the PVA/SA precursor could be determined through the cone-and-plate viscometer, and a variety of experimental parameters were shown in Table S1. The relationship between SEM images of nanofibers and viscosity is shown in Figure 1 and Table S1. Thus, the ESP precursor (5 wt% PVA) with 93 centipoises (cP) demonstrated the microbeads structure or knot-like structures of the nanofiber shown in Figure 1a. Since the viscosity value of the ESP precursor solution was too low, the electrospinning process was subjected to electric field electrostatic force traction. The jet at the front end of the needle tip was associated with disordered behavior due to the low intermolecular cohesion in the ESP solution that made the electrospun fibers less smoothly drawn. Thereafter, the viscosity of ESP precursor fluid was adjusted to 198 cP (6 wt% PVA) and 355 cP (7 wt% PVA) (Figure 1b,c), and the apparent morphology of the electrospun fibers was relatively complete without any defect structure because the electric-field induced traction force reached an equilibrium state to the cohesion of intermolecular chains in the precursor solution. Moreover, the cohesive force of molecular chains was higher than the electrostatic force as the viscosity of the precursor fluid reached 734 cP (8 wt% PVA) (Figure 1d). It might not be easy to obtain the uniformity of nanofibers since the precursor was coagulated and accumulated at the ESP needle tip. Therefore, the average diameter of nanofibers was 92 ± 19 nm, 177 ± 38 nm, 199 ± 22 nm, and 151 ± 22 nm, corresponding to the viscosity of 93 cP, 198 cP, 355 cP, and 734 cP, respectively.

Principally, the SA molecule is chosen as an ingredient of the precursor with high electrical conductivity, and sodium ions affects the fiber’s diameter and morphology. Thus, the volume of the SA solution addition was 0.5, 1, and 1.5 mL to test the appropriate condition for the nanofibers. The viscosity changed accordingly to a variety of SA solution addition. The viscosity of 432 cP, 355 cP, and 295 cP had been determined by 0.5, 1, and 1.5 mL of SA addition, respectively (Table S2). The corresponding SEM images are shown in Figure S1, and the morphology of nanofibers associated with 0.5 mL and 1.5 mL of the SA addition was presented with lots of embedded bead-like structures around the nanofibers due to the instability of the solution jet. The uniformity morphology of the PVA/SA nanofibers occurred in a 1.0 mL SA solution (2 wt%, Figure S2b). As a result, the optimized process condition of PVA/SA nanofibers was associated with the ESP solution viscosity of 355 cP (7 wt% PVA, 1 mL of 2 wt% SA) and with an average fiber diameter of 199 ± 22 nm.

### 3.2. PVA/SA/GO Electrospun Nanofibers

**Figure 2:**
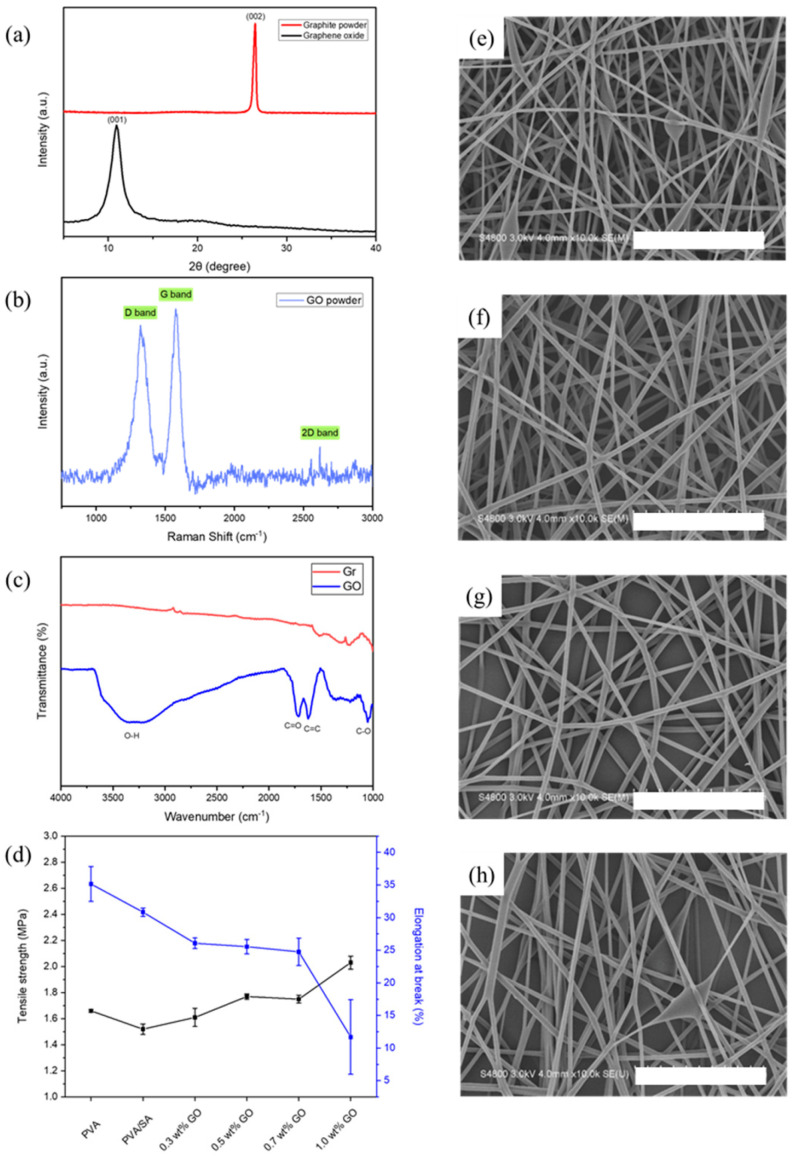
The testing results of (**a**) XRD, (**b**) Raman, and (**c**) FTIR patterns of graphene oxide (GO) via the Hummers’ method. (**d**) The tensile strength and elongation during the breaking of PVA/SA/GO nanofibers. The SEM image of PVA/SA/GO nanofibers with varying GO concentrations of (**e**) 0.3 wt%, (**f**) 0.5 wt%, (**g**) 0.7 wt%, and (**h**) 1.0 wt%, respectively. (The scale bar in the SEM image is 5 μm).

The PVA/SA nanofiber as dressing materials for wound treatment may unfortunately lack thenecessary toughness and mechanical strength. In this study, we tended to employ inorganic graphene oxide (GO) as the ESP precursor to further enhance the mechanical strength of PVA/SA nanofibers. Generally, GO was commonly synthesized by the Hummers’ method with higher oxygen content. The Hummers’ process was followed by the oxidation reaction of high-purity graphite (Gr) and high-strength oxidant. The characterization of GO samples was shown in Figure 2a, the XRD diffraction patterns and carbon interlayer spacing of GO samples were calculated according to Bragg’s Law, as listed in the following equation:

Nλ = 2dsinθ(4)

where N is an integer, λ the wavelength of the incident wave of the Cu target (0.154 nm), *d* the interlayer spacing in the atomic lattice, and θ the angle between the incident wave and the scattering plane. Figure 2a depicts the XRD analysis pattern of Gr with a characteristic peak at 26.5°, indicating the (002) orientation of carbon corresponding to the JCPDS card No. 08-0415, which confirmed that Gr belongs to the hexagonal crystal phase. Then, the carbon layer spacing (d) of Gr was calculated to be 3.36 Å. The characteristic peak of the XRD analysis pattern of GO shifted from 26.5° to 10.9°, indicating the (001) orientation of carbon exists. Referring to the JCPDS card No. 82-2261, the GO sample was validated to be of the cubic phase, and its layer spacing of GO was calculated as d = 8.07 Å. In the case of graphite oxidation, many oxygen-containing groups were sandwiched between the carbon layers, resulting in an obvious expansion in the interlayer spacing of graphite, which suggests that the GO produced by our Hummers’ process did experience a highly oxidized process.

The curve of Raman spectroscopic analysis for the GO sample is demonstrated in Figure 2b. The main characteristic peaks of GO specimens were associated with the D-band, G-band, and 2D-band fingerprints corresponding to their wavelengths located at 1330 cm^−1^, 1580 cm^−1^, and 2670 cm^−1^, respectively. The existence of the characteristic peak of the D-band represents that there are several possible dislocations, lattice voids, defects, or lowering of the area of the sp^2^ structure in the carbon material. The ratio of Raman spectral intensity of D-band and G-band (I_D_/I_G_) was used as a simple and facile indicator for judging the number of defects in the graphite structure. The I_D_/I_G_ ratio of our production of the GO sample was 0.91, which signifies there are some crystal defects and sp^2^ carbon atoms in the GO powder specimen. Moreover, the FTIR was used to measure and analyze the functional groups of GO and Gr (Figure 2c). Prior to the FTIR measurement, we placed both GO and Gr samples in an oven for 24 h to ensure the existing moisture would not affect the subsequent FTIR results of such samples. Our FTIR analysis results show that there were only some absorption peaks, reflecting the existence of Gr. Those FTIR peaks were directly connected to a relationship with the carbon raw material bearing several functional groups. When the Gr sample was strongly oxidized and transformed into GO powder, the original O–H (wavelength at 3300–3600 cm^−1^) characteristic absorption peak of the sample became more obvious, such as C = O (wavelength at 1670∼1780 cm^−1^), C = C (wave number 1650–1675 cm^−1^), C-O (wave number at 1050∼1150 cm^−1^), coupled with some other characteristic absorption peaks also naturally appeared. Therefore, the results of the FTIR analysis confirmed that we successfully transformed the graphite material into a high-purity GO sample.

After validating the GO properties, the PVA/SA/GO precursor solution was prepared by electrospinning with varying GO concentrations of 0.3 wt%, 0.5 wt%, 0.7 wt%, and 1.0 wt%, respectively. During the 0.3 wt% GO solution addition, the non-uniformity and bead-like structure of PVA/SA/GO nanofibers generation because of less charge density of precursor led to the non-stable electrostatic force (Figure 2e). The GO concentration increased to 0.5 wt%, and the high-quality and uniform PVA/SA/GO nanofibers were obtained because the electrostatic force reached a stable state (Figure 2f). While raising GO concentration up to 0.7–1.0 wt%, a higher concentration of GO aqueous solution would accompany an enormous density of charge of the precursor liquid, allowing the ESP jet to be dragged by an applied electric field. As the electrostatic force instantaneously increased, an unstable jet of the precursor liquid around the electrospinning needle was exerted. Thus, PVA/SA/GO nanofibers became relatively non-uniform with pronounced surface-roughness, as illustrated in Figure 2g,h. Furthermore, the optimized PVA/SA/GO nanofibers were determined by a mechanical strength analysis. Following the tensile strength and elongation test results in Table S3 and Figure 2d, the PVA and PVA/SA nanofibers without GO addition showed individual tensile strength of 1.66 MPa and 1.52 MPa. In contrast, the tensile strength gradually increased to 1.61, 1.77, 1.75, and 2.03 MPa featuring GO concentrations of 0.3, 0.5, 0.7, and 1 wt% addition, respectively. Based on the tensile strength results, the maximum enhanced 34% of tensile strength; however, the elongation at breaking suddenly dropped down to 11% during 1 wt% GO addition. We speculated that the 1 wt% of GO addition was distributed as a two-dimensional planar texture, some of the GO might be perpendicular to the longitudinal direction of the nanofiber. Under the external stresses, the additional interaction between PVA, SA, and GO would promote the bonding of molecular chains, leading to the limited activity of GO and stress distribution. Among them, the elongation at breaking gradually diminished after the GO dopant was introduced in the nanofiber system. As a consequence, the optimized condition of PVA/SA/GO nanofibers was associated with the tensile strength of 1.77 MPa (7 wt% PVA, 1 mL of 2 wt% SA, and 0.5 wt% GO) and with the elongation of 25.54 ± 1.1%.

### 3.3. ZnO Nanomaterials

PVA/SA/GO nanofibers possessed both high quality and toughness dressing properties. Consequently, the ZnO nanomaterials were used to implement the objective of an antibacterial agent. The 0.5 M of Zn(NO_3_)_2_ was mixed with various concentrations of NaOH to prepare ZnO nanomaterials via the precipitation method. Hereby the chemical reactions involved were depicted as following equations [30]:

(5) $$\mathrm{Zn}{\left({\mathrm{NO}}_{3}\right)}_{2}+2\mathrm{NaOH}\to \mathrm{Zn}{\left(\mathrm{OH}\right)}_{2}+2{\mathrm{NaNO}}_{3}$$

(6) $$\mathrm{Zn}{\left(\mathrm{OH}\right)}_{2}\overset{∆}{\to }\mathrm{ZnO}+H_{2}{O\;}_{\;}$$

**Figure 3:**
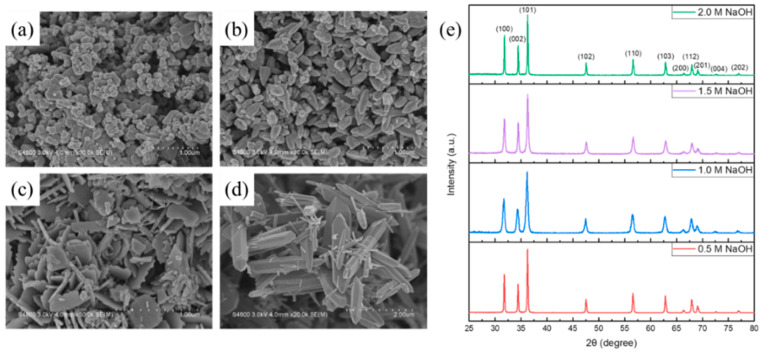
The SEM images of prepared ZnO nanomaterial were obtained by adjusting the NaOH concentration: (**a**) 0.5 M, (**b**) 1.0 M, (**c**) 1.5 M, and (**d**) 2.0 M, respectively. (**e**) the XRD diffraction patterns of ZnO are prepared by preparing different NaOH concentrations.

Interestingly, the SEM images represented a diverse morphology and size of ZnO NPs according to varying NaOH concentrations from 0.5 M to 2 M (Figure 3). Under the lower alkaline concentration, the morphology of the ZnO demonstrated a non-uniform distribution of particle size (Figure 3a). Raising the alkaline concentration, the morphology of ZnO was transferred to the hexagonal (Figure 3b), the plate-like shape (Figure 3c), and the rod shape (Figure 3d), respectively. Based on the variety of the ZnO nanomaterials, we further analyzed these data in detail with an XRD pattern, as shown in Figure 3e. The XRD characteristic peaks of those ZnO samples with the standard data of JCPDS Card No. 36−145−1, where crystal planes of (100), (002), (101) were present together with diffraction peaks indicating (102), (110), (103), (200), (112), (201), (004) and (202) planes. Therefore, the prepared ZnO nanomaterials were determined to be the hexagonal wurtzite structure and they were not involved with impurity diffraction peaks. The average grain size of ZnO was further calculated using the following Scherrer equation:

(7) $$D=\frac{K\lambda }{\beta cos\theta }$$

where D is the average grain size, K was a constant of about 0.9 (varies according to different crystal shapes, the K value range is in the range of 0.89–1), λ the wavelength of X-ray, θ the Bragg diffraction angle, the β was the peak -to-peak width at half maximum of 2θ diffraction in the XRD pattern. When the full width at half maximum (FWHM) of the diffraction peak turned narrower and the peak intensity was enhanced, the average grain size seemed coarser. Conversely, the wider the FWHM of such a diffraction peak and the lower the diffraction peak intensity, the finer size of the grains can be obtained.

The grain size of the prepared ZnO nanomaterials was calculated by the Scherrer equation to 50, 23, 30, and 64 nm with 0.5, 1, 1.5, and 2M NaOH, respectively. The width of the characteristic peak of ZnO samples became narrower, which meant that the grain size increased with an increase in NaOH concentration. However, it was somewhat inconsistent with the trend of our observed experimental results. According to the chemical equilibrium equation [31] represented by the following Equation (8), as the molar ratio of Zn^2+^ and OH^–^ was 1:2, then its chemical reaction could reach equilibrium. However, when the molar ratio of Zn^2+^ and OH^–^ was 1:1, the OH^–^ of this situation could not simply satisfy the complete reaction of Zn^2+^ ions. Thus, the concentration of NaOH was 1.0 M, in turn, this process parameter could lead to the smallest size of ZnO powder.

(8) $$Zn^{2+}{\;}_{\left(\mathrm{aq}\right)}+2OH^{-}{\;}_{\left(\mathrm{aq}\right)}\to ZnO_{\left(s\right)}+H_{2}O_{\left(l\right)}$$

Firstly, we performed the disk diffusion method by using the prepared ZnO nanomaterials for the antibacterial test of *S. aureus*. The experimental results demonstrated the obvious zone of inhibition (ZOI) to indicate excellent antibacterial performance within those four types of ZnO nanomaterials (Figure S3). Generally, the bacteriostatic rings were referred to as the boundary circle corresponding to the inhibiting bacteria efficiency. The ranges of ZOI of 18∼20 mm were contributed by prepared ZnO nanomaterials with 0.5 M, 1 M, 1.5 M, and 2.0 M NaOH treatment (with an average particle size ranging from 30 to 65 nm). In addition, the ZOI of *S. aureus* in 1 M NaOH-treated ZnO NPs (particle size in 23 nm) was slightly larger than other specimens and appeared to efficaciously create a much-pronounced effect on inhibiting the growth of microorganisms. [32,33]

### 3.4. PVA/SA/GO/ZnO Electrospun Nanofibers and the Antibacterial Test

**Figure 4:**
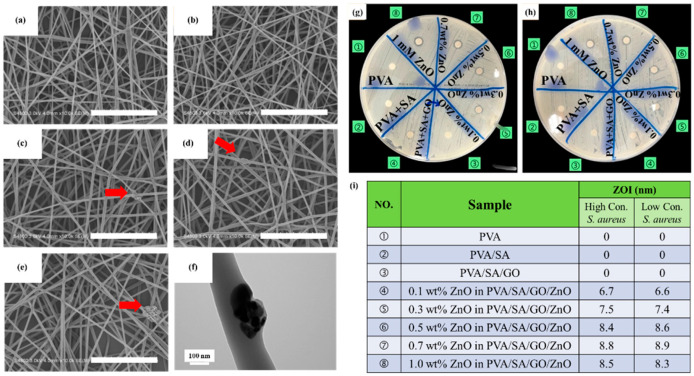
The SEM images of PVA/SA/GO/ZnO nanofibers were prepared by adjusting the amount of ZnO addition: (**a**) 0.1 wt%, (**b**) 0.3 wt%, (**c**) 0.5 wt%, (**d**) 0.7 wt%, with (**e**) 1.0 wt%. (**f**) the magnification SEM image of PVA/SA/GO/ZnO nanofibers with 1 wt% ZnO NPs addition. The antibacterial results of PVA/SA/GO/ZnO antibacterial solution, in which the differences of (**g**) high concentration *S. aureus* and (**h**) low concentration *S. aureus* were compared. (**i**) The zones of inhibition (ZOI) of PVA/SA/GO/ZnO antibacterial solution against *S. aureus*. (The scale bar in the SEM image is 5 μm).

Correspondingly, the 1 M NaOH-treated ZnO NPs (avg. particle size in 23 nm) was employed to fabricate PVA/SA/GO/ZnO nanofibers. The SEM images of PVA/SA/GO/ZnO nanofibers with 0.1∼1 wt% of ZnO NPs addition are shown in Figure 4a–e). After 0.1 and 0.3 wt% ZnO NPs addition, there was no obvious change in the appearance of the fibers (Figure 4a,b). Thereby, the difference in PVA/SA/GO/ZnO nanofibers indicated that a small proportion of ZnO NPs were embedded onto the surface of the electrospun fibers, which caused potential damage to the nanofibers during the amount of ZnO additives up to 0.5–0.7 wt% (Figure 4c,d). Eventually, the ZnO NPs were added to 1.0 wt%, and the PVA/SA/GO/ZnO nanofibers revealed the appearance of ZnO NPs surrounding fibers and generated aggregation illustrated in Figure 4e,f. In brief, the PVA/SA/GO/ZnO nanofibers with the excessive amount of ZnO NPs addition affected the viscosity of the electrospinning precursor and then gave rise to the spinning jet in the ESPs. Therefore, the PVA/SA/GO/ZnO antibacterial solution was demonstrated as an antibacterial solution for *S. aureus* by the disk diffusion method. The antibacterial conditions of the various concentrations of *S. aureus* to the PVA/SA/GO/ZnO antibacterial solution within a series of 1 M NaOH-treated ZnO NPs (0.1–1 wt%) are shown in Figure 4i. As shown in Figure 4g,h, the antibacterial solution without ZnO NPs as test number (No.) ①–③ showed no ZOI for both concentrations of *S. aureus* treatment. For Nos. ④–⑧ of PVA/SA/GO/ZnO antibacterial solution revealed that the more ZnO NPs added, the larger range of antibacterial circles (ZOI) exhibited. According to the antibacterial effect, the antibacterial results directly proved that the PVA/SA/GO/ZnO nanocomposites certainly reveal a significant antibacterial performance. It can be achieved with evidence of an antibacterial ring test for the design of dressing application, the ongoing experiment will be carried out with the PVA/SA/GO/ZnO nanofibers experiments to build up an in vivo medical testing model which will be included in our future report.

### 3.5. Glutaraldehyde Vapor Treated PVA/SA/GO/ZnO Nanofibers

**Figure 5:**
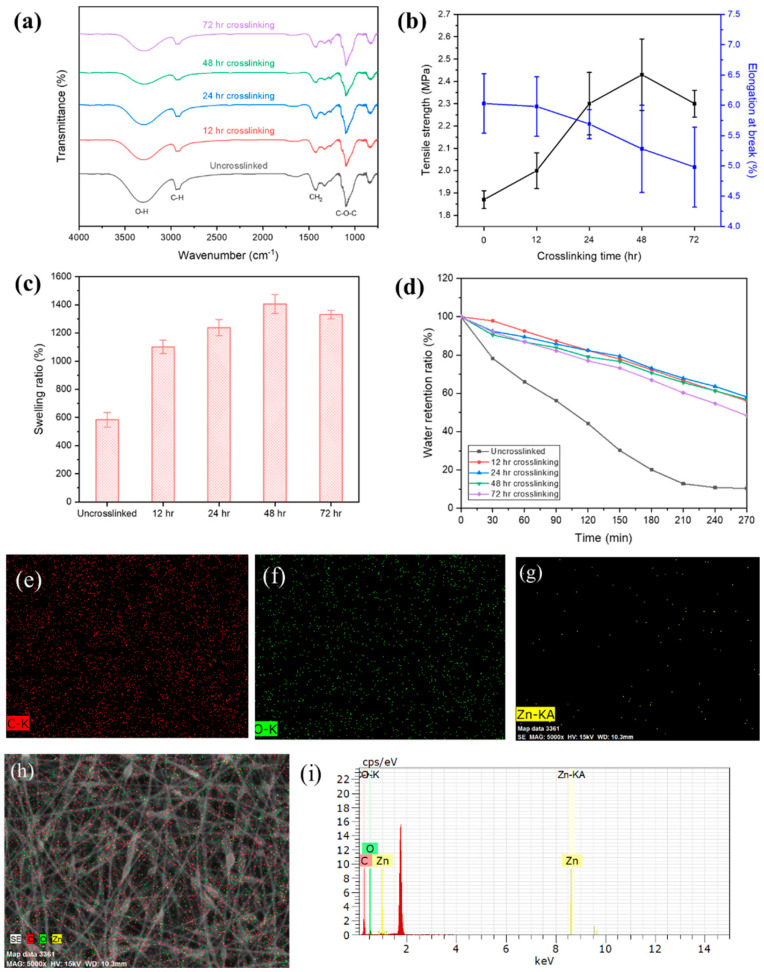
(**a**) The FTIR spectrum of PVA/SA/GO/ZnO nanofibers under different crosslinking time parameters. (**b**) Tensile strength and elongation at break of crosslinked GA-treated PVA/SA/GO/ZnO nanofibers. (**c**) The swelling ratio of GA-treated PVA/SA/GO/ZnO nanofibers within the time course. (**d**) GA-treated PVA/SA/GO/ZnO nanofibers’ water retention capacity rate with different cross-linking times. The EDS mapping of PVA/SA/GO/ZnO nanofibers via 12 h of GA vapor cross-linking reaction: (**e**) carbon, (**f**) oxygen, (**g**) zinc, (**h**) combination of the element mapping image (carbon, oxygen, and zinc) and SEM image and (**i**) the EDS spectrum.

To deal with a practical challenge in which the PVA as an adhesive agent in PVA/SA/GO/ZnO nanofiber regarding self-dissolving in water, the issue encounters nanofibers fused and loss of the original nanofiber structure, therefore, we came up with a strategy to tightly crosslink the PVA/SA/GO/ZnO nanofiber by using the vapor glutaraldehyde (GA) as a crosslinker due to the fact the GA at the liquid phase is naturally volatilized into the gas phase under ambient conditions. Then, the hydroxyl (–OH) group of PVA and the aldehyde group (–CHO) of GA were conducted by the crosslinking reaction, thereby improving the stability and maintaining the morphology of the PVA polymer in water without any specific sticking, dissolving, or fusing. Based on our experimental results, the appropriate parameters used to combine with vapor–GA crosslinker featuring characterizations with FTIR analysis, tensile testing, swelling properties, and water retention analysis (Figure 5). For the FTIR analysis illustrated in Figure 5a, the absorption peak of PVA at the wavenumber of 3500–3200 cm^−1^ represented to O–H stretching vibration of hydroxyl, 3000–2840 cm^−1^ represented the peak of C–H stretching vibration, 1420–1400 cm^−1^ represented the C–H bending vibration of CH_2_, and the stretching vibration of C–O–C near 1096 cm^−1^. For the related characteristic absorption peaks of the cross-linking molecule between GA and PVA content, we could observe in the range of 3500–3200 cm^−1^, where the intensity in 72 h of cross-linking was slightly higher than 48 h [34]. For the tensile strength shown in Figure 5b and Table S4, the tensile strength of PVA/SA/GO/ZnO nanofibers was 1.87 MPa, which was involved with a 5.6% improvement in tensile strength of PVA/SA/GO nanofibers. Furthermore, the tensile strength of GA-treated PVA/SA/GO/ZnO nanofibers was increased to 2.00 MPa, 2.30 MPa, 2.43 MPa, and 2.30 MPa corresponding to the cross-linking time at 12 h, 24 h, 48 h, and 72 h, respectively. The experimental results supported that the time for cross-linking had an apparent tendency to enhance the tensile strength of the samples.

A significantly increased swelling ratio and water retention performance after GA-treated PVA/SA/GO/ZnO nanofibers are shown in Figure 5c,d. The swelling ratio for the sample without crosslinking treatment was 584%, and it increased to 1.101%, 1.238%, and 1.406% after GA vapor cross-linking reaction within 12 h, 24 h, and 48 h. The 48-h GA-treated PVA/SA/GO/ZnO nanofibers exhibited the highest swelling ratio performance and were 1.4 times enhanced than uncrosslinked PVA/SA/GO/ZnO nanofibers. The reasons for the enhanced swelling rate of GA-treated PVA/SA/GO/ ZnO nanofibers are in conjunction with the tightly structured nanofibers that might provide stable porous behavior, and that the SA component played a role in obtaining high hygroscopicity. Furthermore, the water retention capacity with and without GA-treated PVA/SA/GO/ZnO nanofibers prepared by a series of drying and weighing processes every 30 min until 270 min at 40 °C was carried out. The result shows that the GA-treated PVA/SA/GO/ZnO nanofibers displayed a stronger water retention capacity than those samples without cross-linking processes (Figure 5d). The moisture content of PVA/SA/GO/ZnO nanofibers dropped to 10% after the 270 min drying process. In comparison, GA-treated PVA/SA/GO/ZnO nanofibers samples could retain the moisture content of around 80% after the drying step for 120 min and slowly drops down to 50% after drying for 270 min. That is, the GA-treated PVA/SA/GO/ZnO nanofibers possessed a satisfactory hygroscopic ability at room temperature and kept good moisturizing abilities for more than 4 h at human skin temperature (ca. 37 °C) in order to confirm the influence of the crosslinking step on the microstructure of ZnO nanomaterials in PVA/SA/GO/ZnO electrospun nanofibers. The EDS spectrum and X-ray mapping shown in Figure 5e,i exhibits the existence of GO and ZnO nanomaterials into GA-treated PVA/SA/GO/ZnO electrospun nanofibers through crosslinking for 12 h. In addition, the EDS spectrum and X-ray mapping presented additives of both GO and ZnO nanomaterials in PVA/SA/GO/ZnO electrospun nanofibers are intact and well distributed after crosslinking for 24 h, 48 h, and 72 h, which are shown in Figure S4, respectively.

**Figure 6:**
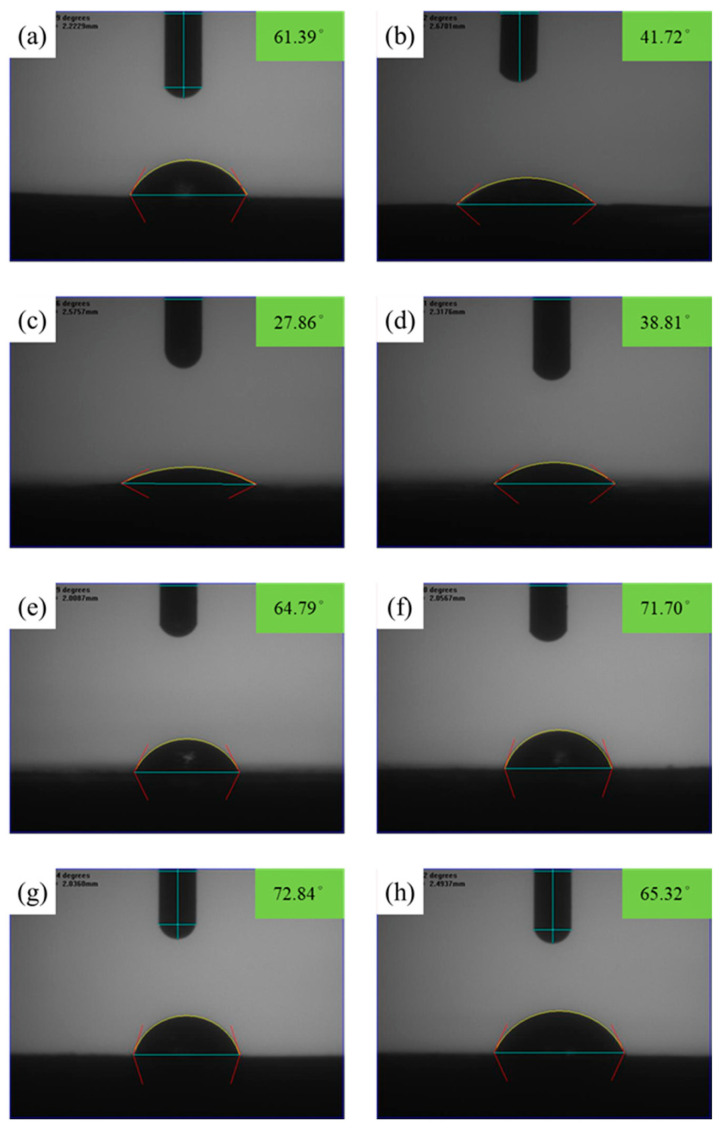
Photographs and calculation of contact angle of (**a**) PVA, (**b**) PVA/SA, (**c**) PVA/SA/GO, (**d**) PVA/SA/GO/ZnO nanofibers, GA-treated PVA/SA/GO/ZnO nanofibers with different crosslinking reaction times: (**e**) 12 h, (**f**) 24 h, (**g**) 48 h, and (**h**) 72 h.

Finally, we investigated the contact angle image of PVA/SA/GO/ZnO nanofibers and GA-treated PVA/SA/GO/ZnO nanofibers to determine the surface hydrophilic/hydrophobic behavior. All the contact angle images and calculation results were shown in Figure 6. The contact angle from pure PVA nanofibers to PVA/SA and PVA/SA/GO nanofibers changed from 61.39° → 41.72° → 27.86°, indicating the SA and GO contribution to the hydrophilicity of the sample surface. Afterward, the contact angle was slightly increased to 38.81° owing to the rough surface with ZnO NPs addition. Thereafter, the contact angle of GA-treated PVA/SA/GO/ZnO nanofibers was evaluated to 64.79°, 71.70°, 72.84°, and 65.32° individually within 12 h, 24 h, 48 h, and 72 h of cross-linking reaction times (Figure 6e,h). Surprisingly, the GA-treated PVA/SA/GO/ZnO nanofibers exhibited an increasing contact angle regarding the cross-linking reaction times raising from 12 h to 48 h, but not to 72 h. Accordingly, the results did illustrate that the GA-treated PVA/SA/GO/ZnO nanofibers could perfectly maintain the hydrophilic surface and present a certain degree of moisture retention.

## 4. Conclusions

In this study, we progressively synthesized the GA-treated PVA/SA/GO/ZnO nanofibers by the electrospinning process (ESP) and successfully investigated their related properties such as nanofiber morphology, mechanical properties, swelling rate, and water retention capacity. Accordingly, the optimized process condition was the combination of 7 wt% PVA, 2 wt% SA, 0.5 wt% GO NPs, and 1M NaOH-treated ZnO NPs (23 nm) with 199 ± 22 nm in diameter, 355 cP in viscosity, and tensile strength of 1.77 MPa. The GA vapor had been used to act as a crosslinker to provide a tight network-like structure and porous-rich nanofiber to support high absorbency and moisturizing for wound healing applications. Interestingly, the GA-treated PVA/SA/GO/ZnO nanofibers displayed a swelling ratio of 1,406% and mechanical strength of 1.87 MPa. Consequently, The PVA/SA/GO/ZnO mixture effectively behaved with an antibacterial ability with an 8 mm inhibition zone in *S. aureus* strains. We strongly believed that such novel electrospun membranes with enhanced water and oxygen permeability would lead to better hemostasis and exudate absorption performance while keeping adequate moisture content in the wound region.

## Supplementary Materials

The following supporting information can be downloaded at: https://www.mdpi.com/article/10.3390/nano13050932/s1, Figure S1: The SEM images of PVA/SA nanofibers with the different added amounts of 2 wt% SA, Figure S2: The SEM images of PVA/SA nanofibers with the different added amounts of 2 wt% SA, Figure S3: The antibacterial test results of Staphylococcus aureus with zinc oxide (ZnO) were prepared with different NaOH concentrations, Figure S4: The EDS mapping (carbon, oxygen, and zinc) and EDS spectrum of PVA/SA/GO/ZnO nanofibers through GA vapor cross-linking reaction within (a,b) 24 h, (c,d) 48 h, and (e,f) 72 h; Table S1: PVA/SA nanofilms made by 8 mL DI solution and their viscosities were detected by cone and plate viscometer, Table S2: Different addition of 2 wt% SA precursors and their viscosities detected by cone and plate viscometer, Table S3: Mechanical properties of PVA/SA/GO fibers with different compositions, Table S4: Mechanical properties of PVA/SA/GO/ZnO fibers with different compositions.
