# Supplementary material for: Fabrication of Glutaraldehyde Vapor Treated PVA/SA/GO/ZnO Electrospun Nanofibers with High Liquid Absorbability for Antimicrobial of Staphylococcus aureus
Source: Nanomaterials (Basel). 2023 Mar 3;13(5):932. doi: 10.3390/nano13050932 (PMC10004850; doi:10.3390/nano13050932)
Supplement: Supplementary file 1 [file nanomaterials-13-00932-s001.zip › nanomaterials-2243023-supplementary.pdf]

Supplementary Information

Fabrication of glutaraldehyde vapor treated PVA/SA/GO/ZnO electrospun nanofibers with high liquid absorbability for antimicrobial of *staphylococcus aureus*

Yi-Hsin Chien[1], Meng-Tzu Ho[1], Chin-Hsign Feng[2], Jung-Hsign Yen[2], Yi-Chan Chang[1], Chih-Sheng Lai[2], Rong-Fuh Louh[*1]

[1]Department of Materials Science, Feng Chia University, Taichung, Taiwan 40724

[2]Division of Plastic Surgery, Taichung Veterans General Hospital, Taichung, Taiwan

[*]Correspondence author: rflouh@fcu.edu.tw

Table S1. PVA/SA nanofilms made by 8 ml DI solution and their viscosities were detected by cone and plate viscometer.

PVA (wt%)	DI water (ml)	SA (wt%)	Voltage (kV)	Work Distance (cm)	Flow Rate (ml/hr)	Viscosity (cP)
5	8	2	15	15	0.5	93
6	8	2	15	15	0.5	198
7	8	2	15	15	0.5	355
8	8	2	15	15	0.5	734

Table S2. Different addition of 2 wt% SA precursors and their viscosities detected by cone and plate viscometer.

PVA (wt%)	2 wt% SA (ml)	Voltage (kV)	Work Distance (cm)	Flow Rate (ml/hr)	Viscosity (cP)
7	0.5	15	15	0.5	432
7	1	15	15	0.5	355
7	1.5	15	15	0.5	295

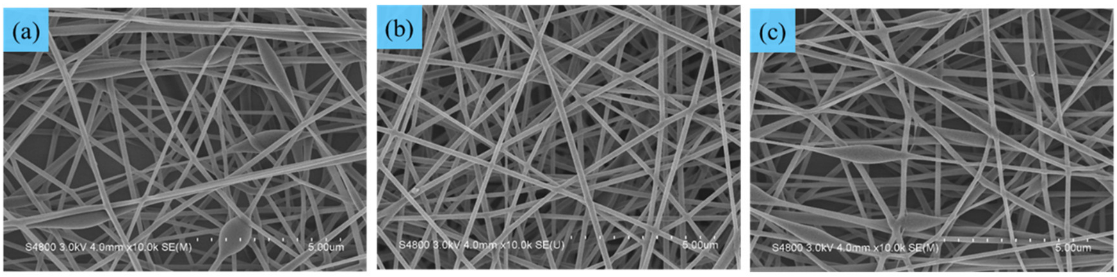

Figure S1. The SEM images of PVA/SA nanofibers with the different added amounts of 2 wt% SA of (a) 0.5 ml, (b) 1.0 ml, and (c) 1.5 ml.

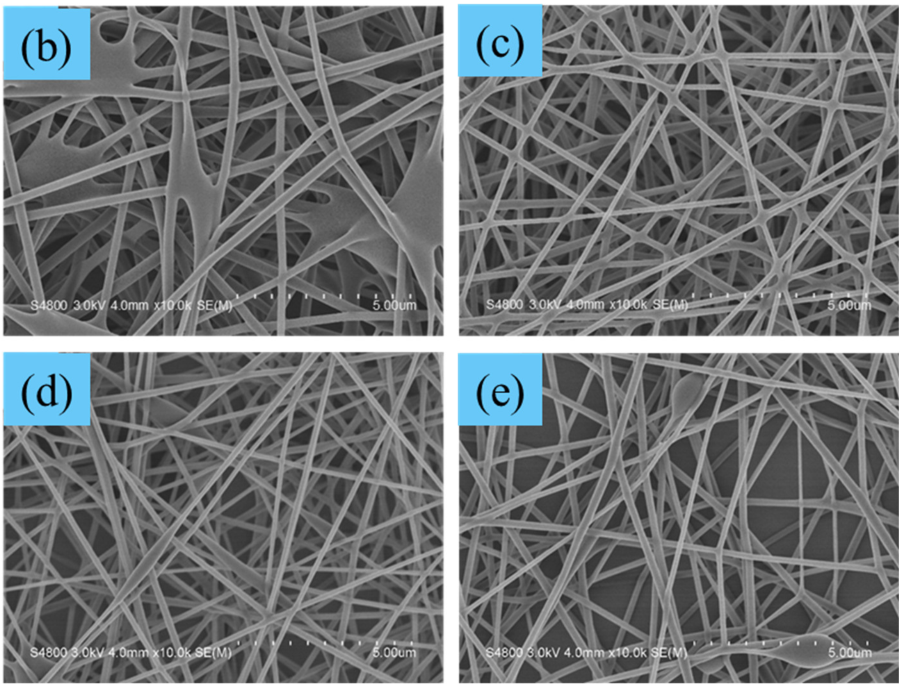

Figure S2. The SEM images of PVA/SA nanofibers with the different added amounts of 2 wt% SA of (a) 0.5 ml, (b) 1.0 ml, and (c) 1.0 ml.

Table S3. Mechanical properties of PVA/SA/GO fibers with different compositions.

	Tensile Strength (MPa)	Elongation before Fracturing (%)
PVA	1.66±0.01	35.16±2.67
PVA/SA	1.52±0.04	30.84±0.64
PVA/SA/ 0.3 wt% GO	1.61±0.07	26.07±0.81
PVA/SA/ 0.5 wt% GO	1.77±0.02	25.54±1.10
PVA/SA/ 0.7 wt% GO	1.75±0.03	24.76±2.09
PVA/SA/ 1.0 wt% GO	2.03±0.05	11.69±5.71

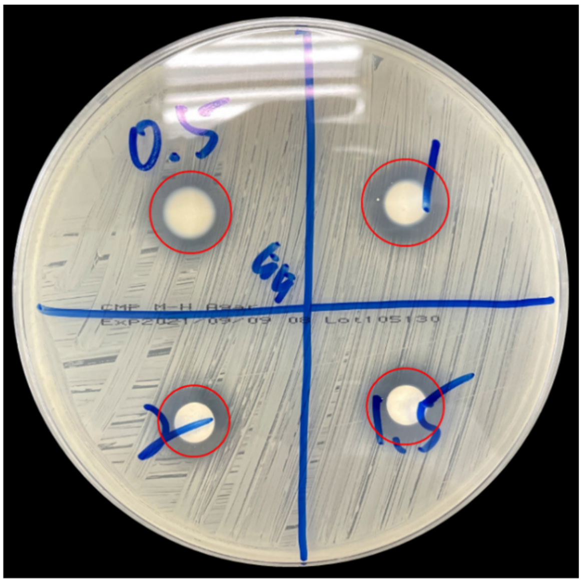

Figure S3. The antibacterial test results of *Staphylococcus aureus* with zinc oxide (ZnO) were prepared with different NaOH concentrations.

Table S4. Mechanical properties of PVA/SA/GO/ZnO fibers with different compositions.

Cross-linking Time (hr)	Tensile Strength	Elongation at Break
	(MPa)	(%)
0	1.87±0.04	6.03±0.49
12	2.00±0.08	5.98±0.49
24	2.30±0.14	5.69±0.24
48	2.43±0.16	5.28±0.72
72	2.30±0.06	4.98±0.66

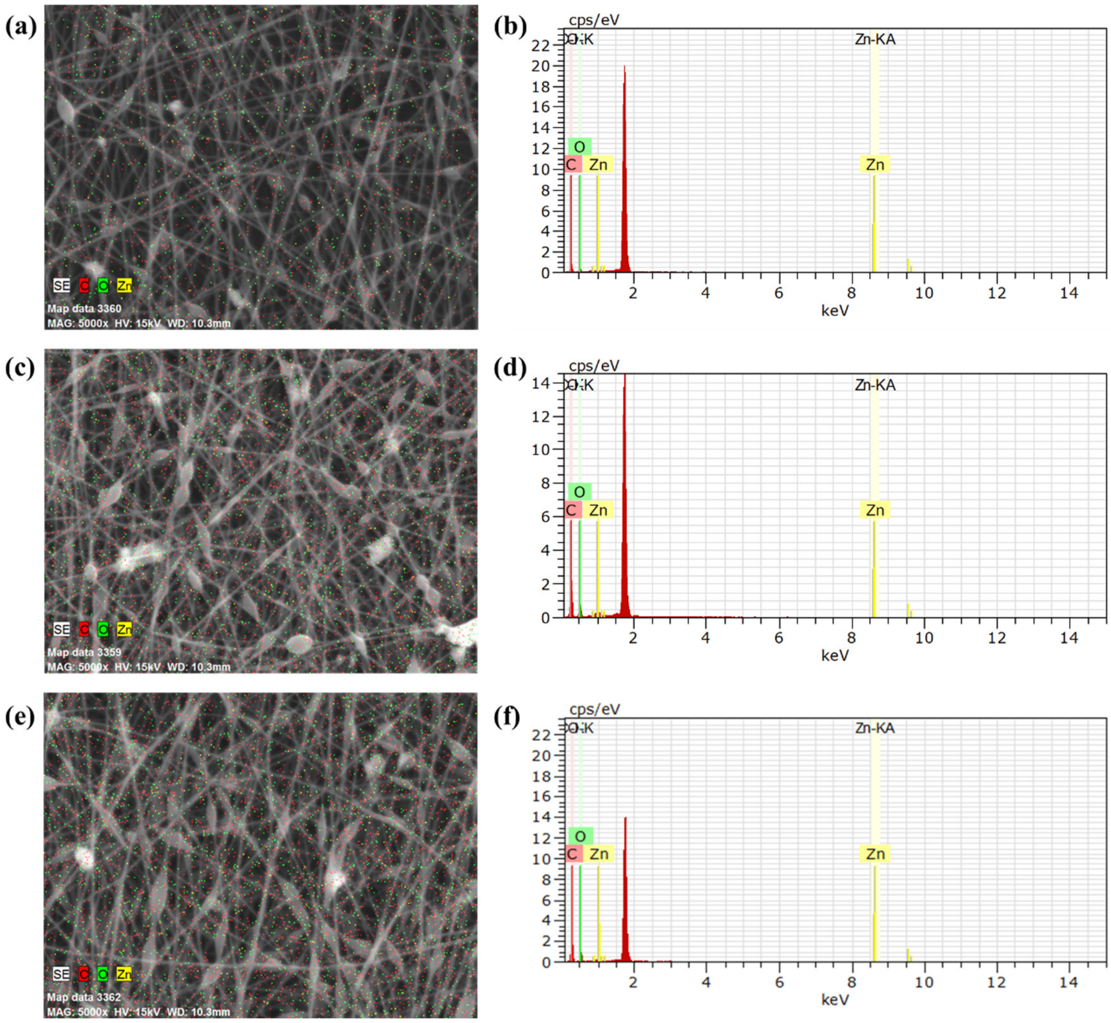

Figure S4. The EDS mapping (carbon, oxygen, and zinc) and EDS spectrum of PVA/SA/GO/ZnO nanofibers through GA vapor cross-linking reaction within (a)-(b) 24 hours, (c)-(d) 48 hours, and (e)-(f) 72 hours.
